# Northeastern Dental Association

**Published:** 1899-04

**Authors:** 


### NORTHEASTERN DENTAL ASSOCIATION.

(Continued from page 126.)

### CAVITY PREPARATION.

BY SIDNEY S. STOWELL, D.D.S., PITTSFIELD, MASS.

Mr. President and Gentlemen,—The subject which I have chosen for this occasion is of more vital importance to the regular practitioner in dentistry, and has more to do with his success or failure, than any other subject in the whole realm of dentistry.

It is my opinion that the man who will so apply himself as to acquire the ability to properly prepare a cavity in a tooth for filling will, as sure as night follows the day, fill that cavity successfully.

I have for eighteen years made this important subject my study. I have availed myself of every possible resource of knowledge. I have exhausted all available literature. I have witnessed the demonstrations of many of the most renowned operators of the world, and have arrived at one grand conclusion, summed up in two short sentences, the truth of which I challenge any one to gainsay: The right way to prepare a cavity is *all right!* The wrong way is *all wrong!*

I suppose, gentlemen, that each one of you knows how to prepare a cavity in a tooth for filling as well as I do, and I suppose I know how as well as you, and yet I venture that no two of us would prepare the same cavity exactly the same; therefore some of us must be wrong. I will state the three most vital principles in cavity preparation which may be accepted as *all right.*

First, free cutting of all frail margins remote as well as on the front.

Second, self-cleansing margins.

Third, avoid trespassing upon the domain of that sensitive member the pulp, numbers 1 and 2, round burs being large enough to make all grooves and anchorages.

(Specimens of cavity preparations were passed around.)

Dr. C. Frank Bliven, of Worcester, Mass., followed with a short article entitled "A Guess."

### A GUESS.

BY C. FRANK BLIVEN, D.D.S., WORCESTER, MASS.

Good guessing is said to be one of the accomplishments of a thoroughbred Yankee. Having been born in Connecticut, I should by birthright be an accurate guesser.

I am going to make a guess! Some day dental colleges will discover that dentists, like poets, are born, not made; that neither the possession of a good intellect nor the possession of degrees will make a dentist. Perception, concentration, courage, patience, endurance, gentleness, firmness, these the dentist must have, and, besides these, tendencies scientific and artistic, with a natural aptitude for his work. When colleges can create these qualities, they can build a dentist out of any man; until then there will be few dentists even among those who write D.D.S. after their names.

Two things are necessary as the basis of success in life,—first, the choice of a profession for which the individual is by taste and tendency adapted; second, such education as will develop his special gift. Who can estimate the tremendous amount of energy wasted daily by those following uncongenial employments? What needless friction would be avoided if men could do the work they liked to do! Not only would all the world's necessary work be done, but it would be done in less time, leaving the worker's leisure hours each day for recreation and higher culture.

Colleges are serving a purpose: they aid the intellectual development of their students and add to their information. Colleges, however, have not yet struck the principle which, when discovered and applied, will insure the success of every earnest student; they do not develop character.

This day of diploma-bearing dentists is also the day of the "dental parlor." Twenty-five years ago there were no "dental parlors." The dentist was a dignified, professional gentleman. Laws have been passed with intent to "elevate the profession." That end cannot be accomplished by the enactment of laws. So long as men who take a given course of studies and pass given examinations are graduated by colleges without reference to special fitness for the chosen profession nor to their moral standing, so long will charlatans be protected in their competition with honest men.

There is one difference between an ignorant man and an educated one,—the latter can do more mischief. An uneducated thief will steal your overcoat or umbrella; an educated thief will steal your house or your business. An uneducated thief is subject to law as it stands; an educated thief will use his power to *make* laws which protect him and his kind.

Colleges will become unqualified powers for good when they not only seek to develop a man's abilities, but seek to develop them for the highest end,—service to his fellow-men. Let dental colleges graduate no men but those especially adapted by nature to the profession and those whose conduct has shown a consciousness of responsibility for the welfare of others. There will then be no further talk of "elevating the profession." The profession will be elevated and the public protected. If not, I shall have to guess again.

*Thursday Morning, 9.25.*

Dr. E. B. Dickinson, of Amherst, Mass., read a paper on "Ethyl Bromide as an Anæsthetic."

(For Dr. Dickinson's paper, see page 91.)

DISCUSSION.

*Dr. G. A. Maxfield* (Holyoke, Mass.).—It seems to me that we should be very conservative in accepting new anæsthetics. We all know that in every anæsthetic there is danger. When under the influence of an anæsthetic, a person is as dead as can be possible in life. Ethyl bromide has not yet been accepted by the medical profession. We should wait until the profession has tested it and pronounced it safe. I really think the use of ethyl bromide will pass out of use. We realize the great trouble and danger in giving these drugs. I have not used one for four or five years, and I always advise patients to employ a physician at their own homes. It relieves me of a great responsibility. In the present condition of affairs the public do place more confidence in these things than physicians. This ethyl bromide was used eighteen or twenty years ago and dropped out of use, and to-day it is revived. But it seems to me that we should wait for the medical profession to adopt it.

Dr. Stockwell expressed agreement with Dr. Maxfield.

*Dr. James McManus.*—I think the paper well presented. But this constant finding fault with the colleges is what I object to. It is a great many years since I attended college, but materia medica was taught then. The students were required to study it. If a student cannot pass his examination, lay the blame on the student, and not on the college. When a student is given a list of the works on materia medica, and does not take advantage of his opportunities and fails in examination, it is the fault of the student. I protest against the constant finding fault with the colleges.

Dr. Flanagan asked if there were not records of cases where ethyl bromide had an evil effect?

*Dr. Dickinson.*—There was one case where ethyl bromide was used by the continuous method, but that is not a common way to use it; forty drachms of the anæsthetic were given; and in the other case—I think I have the case in mind which the gentleman refers to—the patient's mouth was covered with sores. I know of those two cases, but they are no detriment to ethyl bromide.

*Dr. Flanagan.*—I want to thank Dr. Dickinson for giving us the paper, for it is out of the ordinary; but, as Dr. Maxfield said, suppose we have a death, we have the authority of America against us, and would have to stand trial.

*Dr. G. A. Mills* (New York).—I have an admiration for men who will emphasize what they mean. I am willing to take this man's word. I am willing that he should use ethyl bromide if he wants to. I am not an expert, but I use cocaine as readily as paregoric, and have absolute success with it. Take two gentlemen whose testimonies absolutely disagree, what are you going to do?

Simply let them go on. Opinion is divided over the world in regard to chloroform, but perhaps the majority are against it. But in Scotland they have the lowest death-rate of any country in the world, and they use chloroform. In Baltimore, Shaker uses it. You know yourself what to do. It is in the man. If a man has that within him which gives him confidence to do these things, that confidence will take care of him. One cannot rush into these things. If you do not agree with a man, do not get out of temper. It does not do to call a man a fool in these days. Let the man demonstrate his ability.

*Dr. Maxfield.*—From the questions that are asked the graduates, you will see that the stress is not laid upon materia medica that is laid upon other studies. It is a branch upon which more stress will have to be laid, because scientific advocates will demand it. I believe in bringing the colleges up to just as high a standard as possible. I am working for this one thing. In regard to the paper of Dr. Dickinson, it has the effect of impressing upon our minds that there is such an anæsthetic coming along, and we will be on the lookout for it. As to criticising the old preparation, I do not care to go into that. The criticism of this is of no account if the new preparation is good. If this drug is what it is claimed to be, it gives relief from pain before consciousness is lost. I am not willing to give up cocaine. I wish I were a young man, that I might study. I have an ambition for the dental profession to be something and have its right place.

*Dr. G. Lennox Curtis* (New York).—I listened to the paper with great interest. The tone of the papers here has been of a much higher grade than is generally heard at such a meeting. My attention was attracted to ethyl bromide in Baltimore, in 1880, by a paper from Dr. Chapin on eye-disease. He spoke there of the importance of the drug and of the danger in using it. I procured some of it and began experimenting, first upon myself. While under its influence, a man is in possession of all his faculties, but pain is gone. Dr. H. C. Wood told me that he believed ethyl bromide to be more dangerous than ether and less so than chloroform. For minor operations I like it very much. Of late I have not been using it, because cocaine has taken its place. The rapidity with which you can operate with ethyl bromide is of great advantage. I remember giving a clinic at one time at which were several who had used ethyl bromide. Some of them, being friends, when I began to operate, said, "Good-by, we will not be present at the death, as we do not want to appear in court." Several left the room. The operation occupied one minute. The patient suffered no pain, but was conscious of having work done. I have had no trouble in the use of ethyl bromide. I have had patients do very funny things. Sometimes they imagine themselves on a trapeze.

I have no right to work with an agent with which I am not familiar. I cannot get familiar with anything except by experimenting. Dr. Dickinson is an expert. Those who have occasion to use such a short-acting anæsthetic should employ some one familiar with it. I do not know that there is a patient here for me. I expected to demonstrate in a work the use of cocaine and the value of the antidote. If there is not a patient here for me, I will give you a talk on surgery.

The report of the Committee on Dental Law was called for, and the following was adopted by a unanimous vote:

It having been brought to the attention of the Northeastern Dental Association that a committee has been appointed by the National Association of Dental Examiners to bring about a uniformity of dental laws throughout the United States, and to arrange for the recognition of the certificate of one State, having a standard examination, by other States, this Association, aware of the importance of the subject, expresses its approval, and trusts the committee will do all in its power to accomplish this object at as early a day as is practicable.

*Resolved,* That the secretary forward this resolution to the chairman of the committee, Dr. William Donnelly, of Washington, D. C., and also to the president of the Northeastern Association of Dental Examiners.

*Resolved,* That the president appoint a committee of one from each State represented in this Association, to give this matter their earnest attention.

*Dr. W. H. Ryder* (Danbury, Conn.).—I do not think that we should pass the paper of Dr. Smith as we did yesterday, with no remarks. I cannot agree with Dr. Smith myself, and I do not think that paper should be accepted without a protest on our part, showing that the society practically accepts his theory of stimulating the teeth by cleansing them. If he will try this pumice-stone on some other part of the body, he may get the required stimulation, but I know from practical experience that it is bad practice to use it on the teeth. It is impossible to get pumice-stone so fine that it will polish enamel on the human teeth. And teeth apparently so polished, even under an ordinary glass, will give you the appearance, on the surface, of ice which has been lately skated upon. The enamel is full of scratches.

Some years ago, when I was young, I carried on a number of experiments to find the action of certain medicines. I tried an experiment on two incisors in the same mouth. One was polished with orange-wood and the finest pumice-stone, and the other not polished at all. I placed both under a microscope. The one which I had not polished presented a smooth, polished surface, and the other was covered with scratches. That was only one of many experiments.

*Dr. Murlless.*—When we have an accumulation on the teeth, and the danger from acids has been created by the decomposition of the accumulation, we are in the position of the man who said the practice of medicine was a choice of two evils.

*Dr. Mills.*—This is one of the most interesting subjects in the profession. You can polish a tooth and make it beautiful.

*Dr. Smith.*—Dr. Ryder has made some concessions which, it would seem, all dentists ought to take notice of. If these materials which are in the hands of every dentist in the land, I suppose, are so wonderfully injurious, so destructive to the enamel, it ought to be published and sent broadcast. If we have made this mistake for all these years, we ought to know of it. I have practised for thirty-two years, and have these materials every day, and have them in use for polishing the teeth. If they have done so much mischief, I ought to have known it and the profession ought to have known it.

Recess.

*Dr. G. Lennox Curtis* (New York).—I have not come prepared to talk on any one subject. I was in hopes of doing some work and letting you see how diseases of the jaw and face are treated, and it is quite a disappointment to me not to be able to do this. There has been but one case brought for examination, a case of antrum disease, which I endeavored to explain. I would like to talk on the subject of cocaine, upon which Dr. Mills desired me to speak. Since I have felt free to use cocaine in any solution, I use it with as much freedom as water. I would not hesitate, and do not hesitate, to use the saturated solution of cocaine in any class of disease, or for any class of operations, where cocaine can be applied with perfect safety to the patient. Only recently a patient whom I know had a very serious surgical operation performed in Baltimore by Dr. Kelly, consisting of an abdominal incision and the walls of the bladder being brought up in contact with the abdomen to sustain it, the person having prolapsus of the bladder. This operation was performed with cocaine, the patient being unable to take an anæsthetic. The surgeon used volasen with the cocaine. He would not for a minute have thought of performing the operation with cocaine alone. The patient wrote me a letter telling me that the operation was performed with perfect safety, and he experienced no pain, though he was conscious. By the use of this antidote, which has been in the market for some time, you can use cocaine with impunity. It is a proprietary preparation, and, of course, physicians would not admit that they use it, but they do, and if they did not they should be condemned. Anything that will do away with the bad effects of cocaine should be used. It stimulates the respiratory and cardiac functions and prevents depression. It is given in from one- to five-drop doses before you inject the cocaine. If, after injecting the cocaine, you notice any functional derangement, repeat the dose. I follow the directions given on the label, and use it with freedom. This volasen is, in itself, a paralyzer, and, without cocaine, a teaspoonful will produce death. I would suppose that where an accident occurred, if you gave cocaine, it would antidote it.

In July, just as I was leaving my office, my house being closed for the summer, a dentist brought a patient for an operation. The patient would not take an anæsthetic. I spoke of this antidote and said that I would use cocaine. As my nurse had gone, I told the dentist that if he would take care of the case, I would operate. I prepared the volasen, pouring out the usual amount, and prepared my cocaine. In preparing cocaine I take a small glass of distilled water and throw in the cocaine without measuring, to get the saturated solution. I filled the syringe with a drachm of the saturated solution. (It was an extensive operation.) I picked up the syringe and went to work. I was just nicely started, when I found my patient crazy. It was all that we could do to hold the man. I remarked that it was a peculiar condition. I reached for the glass to give him more volasen, when I found that I had not given the volasen at all. Two minutes after giving him this, he was all right. That showed conclusively the power of the drug and the benefit. So I have suggested it for cocaine-poisoning where great mistakes of that kind have been made and two or three drachms of a ten-per-cent. solution of cocaine given. I do not believe it is safe to wait; it should be given in advance of cocaine. I have never used this hypodermically. I imagine it would act more promptly. I will tell you of one or two cases in practice which show neglect on the part of the dentists, or else a lack of knowledge of how to diagnose abscessed teeth, and the great need for teaching in this line. In dentistry, as in medicine, most of the colleges are independent institutions, and they are organized to help a few men (if you will forgive me for speaking against the colleges); but I want to say that the heads of colleges will rarely put in a man who can demonstrate the value of new ideas. They want the same old men to stay and die in the colleges, when their places might be filled by hundreds of young men who are in poverty because of their love for science. Our colleges ought to put in men such as are found in the dental profession to-day. And there are men here who are thoroughly competent to teach pathology.

Here is the case of a woman, fifty-seven years of age, who for forty years had a swelling on the neck. This had been opened every year, with a discharge of pus. The physician said the disease was of the thyroid gland, of which it is said that a one-sixth part must always remain or the patient will be crazy. The first surgeon probably made this remark, and it was told to all the others, so they kept clear. Four weeks ago the physician called on me, told me of the case, and asked if I would see the patient. I remarked that, aside from the operation in the neck, the dentist could do the work. I told him that the trouble was an abscessed tooth. He replied that her teeth were perfect. I said, "I believe we will find a decomposed pulp." The teeth looked clear and bright and healthy, but on percussion, when I reached the lower bicuspids, I found that the sound was not the same. It was a leaden thud, which indicated an unhealthy condition. I explained that these teeth had decomposed pulps, and told the lady to go to the dentist and have them cleaned. She reluctantly followed my instructions, and later came into the office. The teeth had been properly cleaned. I operated on this case, and removed fully three ounces of pus from the cavity. It was a fistula. The patient's health is about restored, fever and pain have disappeared. She had been undergoing a surgical operation and was sick for some time every year. That case shows oversight.

Another case, which came under my notice in September, was that of a man who was seized with such a pain in his head that he fell to the ground. The policeman took him to the lock-up, a safe place. After a while an ambulance was summoned, and he was taken to Bellevue Hospital and placed in the insane ward. He was afterwards placed in the insane asylum and stayed there two years. At the expiration of the two years he received a certificate stating that he was not insane, and was a proper subject for association with other people. I diagnosed the case. In percussing the teeth, as I reached over and struck the upper right first molar, I noticed a curve of the head. That was all. I hit it hard. The man remarked that it hurt. I sent him to his dentist to have the tooth extracted. On breaking the tooth open, it was found to contain pulp-stones. To this day he has not had a return of the pain. His business had been ruined because of the ignorance of the physician. Such cases are coming along all the time, where dentists are the best workers and medical men know nothing of the case.

In reply to the question concerning hyperæmia from pulp-stones: My principal way of diagnosing a case is by percussion. Any abnormal sound indicates disease. If there is a higher pitch note, I look for pulp-stones. The treatment is the same. The pain is rarely ever found in the teeth; it is in the brain or upper part of the head. In those cases of long-continued neuralgia which have resisted everything, you can generally look for the cause in the teeth. About ninety per cent. of these diseases come from the teeth. In fact, all diseases of the jaw and face are the result of affections of the teeth. The soreness or tenderness will indicate disturbance there. You can often find the place by electricity: apply it to a solid tooth, and you get a shock; also by the hot and cold water test, which is the poorest method of all. The ordinary test is of very little value. It is the knowledge gained by seeing these cases which tells. Diagnose your case by going from one tooth to another. If the pain is on one side, examine that side, tooth by tooth. Some people are much more responsive than others.

*Dr. Codman* (Boston, Mass.).—When the teeth are struck, do I understand that the pain is inside the teeth or beyond?

*Dr. Curtis.*—Beyond the teeth, rarely in them; if so, an exception.

*Dr. Codman.*—How is it that the tooth which has pulp-stones always lacks an articulating tooth?

*Dr. Curtis.*—I believe every tooth can be in the head and articulation perfect, and still have pulp-stones.

*Dr. L. W. Fowler.*—I have a case here of a man about nineteen years of age. His teeth were perfectly even, the articulation perfect, the teeth all right so far as my judgment went. He had some trouble in the left side of his head. He had no idea that there was anything the matter with his teeth. He followed his physicians' directions. They prescribed drug after drug. After a while he gave up his business. He happened to be a brother-in-law of mine, and his case came under my notice. After he left his work I began to look up the case myself. I found that when the paroxysms came, the left eye would sink from the intense pain. He slept off from my room. One morning I awoke when he was in a paroxysm. He resembled a person with horrors, and expressed himself by saying, "Leave me. Leave me. I fell out of the window and broke in two, and you cannot do anything for me." I drew my attention to his teeth on the left side. I noticed a marked difference in percussion in the teeth on the left side. I told him if he noticed any soreness in a certain tooth, to come to my office. In about two days, after he had passed through another paroxysm, he came to my office. I removed the tooth. That was seven years ago, and he has had no trouble since.

*Dr. Codman.*—This articulation of the tooth took place long before the tooth had been extracted. Nature had been trying to obliterate the pulp.

*Dr. Stowell.*—I am positive that I have found pulp-stones where occlusion was normal. I can recall three such cases where occlusion was good and the teeth were perfect.

*Dr. S. B. Palmer.*—I cannot say that I can add anything. I had one case in particular. The patient was confined to a rolling-chair by rheumatism. She had paroxysms from one side of the face. The teeth were apparently in perfect condition. The paroxysms came at meal-times, and she was almost frantic with the pain. I concluded to extract the first superior molar. There was no choice for treatment, and I decided to extract the tooth, which I did and took it home. Instead of cutting it open, I ground it down and found the cavity filled with pulp-stones. There was no more trouble after the removal of the tooth. I have had several cases, but none so marked as that one. There is more trouble arising from the irritation of the pulp than we are aware of. In some cases trouble will arise in the clasped teeth, and in cutting them open you will find pulp-stones on the opposite side, as though nature had attempted to form some barrier and had filled the canals and the pulp-cavity.

The following officers were elected: President, Clinton W. Strang, Bridgeport, Conn.; First Vice-President, G. F. Harwood, Worcester, Mass.; Second Vice-President, A. J. Cutting, Southington, Conn.; Secretary, Edgar O. Kinsman, Cambridge, Mass.; Assistant Secretary, F. M. Wetherbee, Milford, N. H.; Treasurer, I. Tenney Barker, Wallingford, Conn.; Librarian, F. T. Murlless, Jr., Windsor Locks, Conn.; Editor, Charles McManus, Hartford, Conn.

Meeting adjourned.

CHARLES MCMANUS,
*Editor.*

HARTFORD, CONN.

### Editorial.

